# The impacts of Ramadan fasting for patients with non-alcoholic fatty liver disease (NAFLD): a systematic review

**DOI:** 10.3389/fnut.2023.1315408

**Published:** 2024-01-17

**Authors:** Xiaoxiao Lin, Guomin Wu, Jinyu Huang

**Affiliations:** Affiliated Hangzhou First People’s Hospital, School of Medicine, Westlake University, Hangzhou, Zhejiang, China

**Keywords:** Ramadan fasting, non-alcoholic fatty liver disease (NAFLD), impacts, liver parameters, a systematic review

## Abstract

**Background:**

Numerous studies have explored the impacts of Ramadan fasting on Non-alcoholic fatty liver disease (NAFLD). Therefore, the objective of this systematic review was to analyze and summarize all clinical studies regarding the impacts of Ramadan fasting for patients with NAFLD.

**Methods:**

We performed a comprehensive search of the Embase, Cochrane, and PubMed databases from inception to September 1, 2023. All clinical studies concerning the impacts of Ramadan fasting on patients with NAFLD were included.

**Results:**

In total, six studies with 397 NAFLD patients comprising five prospective studies and one retrospective study were included in the systematic review. All six studies were assessed as high-quality. Ramadan fasting may offer potential benefits for patients with NAFLD, including improvements in body weight, body composition, cardiometabolic risk factors, glucose profiles, liver parameters, and inflammation markers.

**Conclusion:**

Ramadan fasting might be an effective dietary intervention for NAFLD. However, the number of studies examining the impacts of Ramadan fasting for patients with NAFLD is relatively limited. Therefore, more high-quality research is needed to further our understanding of the benefits of Ramadan fasting for NAFLD.

**Systematic review registration:**

https://inplasy.com, identifier 202390102.

## Introduction

Non-alcoholic fatty liver disease (NAFLD) encompasses a spectrum of liver conditions, ranging from simple steatosis to non-alcoholic steatohepatitis (NASH), and can progress to more severe forms such as cirrhosis and hepatocellular carcinoma (HCC), which is defined by the presence of fat in more than 5% of hepatocytes, and not attributed to the causes such as viral hepatitis, excessive alcohol consumption, steatogenic medications, or other causes of fatty liver disease (1, 2). Its development is influenced by an interplay of genetic predisposition and environmental factors. A recent study found that the global incidence of NAFLD is 4,613 per 100,000 person-years, with a higher incidence among overweight or obese individuals, and men (3). It is estimated that the global prevalence of NAFLD is 25%. NAFLD poses a significant public health challenge worldwide. So far, there is no approved drug therapies for NAFLD. The complex pathophysiology and substantial heterogeneity in its phenotypes suggest that combination treatments might be necessary. For the prevention and treatment of NAFLD, a healthy lifestyle is of vital importance.

A balanced diet is the main component for those with NAFLD and is endorsed by current guidelines. Numerous studies have shown that specific dietary interventions can positively affect liver and metabolic parameters in patients with NAFLD. These interventions include the Mediterranean diet (4), time-restricted eating (TRE) (5, 6), low-carb diets (7), alternate day fasting (ADF) (8), the 5:2 diet (9), and Ramadan fasting. Among them, Ramadan fasting is a unique form of obligatory intermittent fasting observed annually during the month of Ramadan by approximately 1.5 billion Muslims worldwide. This fasting regimen offers various health benefits such as improved body weight and composition, reduced complications from metabolic syndrome, enhanced lipid profile, and other cardiometabolic advantages. Furthermore, Ramadan fasting fosters better glucose homeostasis, reduces inflammatory and oxidative stress markers, and may influence gene expression associated with anti-inflammatory and antioxidant defenses. Previous studies have investigated the effects of Ramadan fasting for healthy adults (10–17), patients with prediabetes and diabetes (18–29), and individuals with NAFLD (30–34). Among them, many studies have delved into the relationship between Ramadan fasting and NAFLD. For example, Badran et al. (30) investigated the effects of Ramadan fasting on 98 patients with NAFLD. They found that Ramadan fasting led to significant improvements in anthropometric biochemical, and ultrasonographic parameters for NAFLD patients, especially in the early phases and among prediabetics. Mari et al. (32) explored the effects of Ramadan fasting on patients with NASH and found that fasting could improve NASH severity, insulin sensitivity, and inflammatory markers. However, no systematic review has been performed to compile these clinical trials. Therefore, the aim of our study is to conduct a systematic review to summarize and analyze all clinical studies regarding the impacts of Ramadan fasting on patients with NAFLD.

## Methods

### Search strategy

We conducted this systematic review in accordance with the Cochrane Collaboration guidelines. Our review protocol was pre-defined and registered on the INPLASY website (ID:202390102), and we have reported the results in line with the PRISMA checklist (35). We undertook a comprehensive search of the Embase, PubMed, and Cochrane databases from their inception up to September 1, 2023, and without language restrictions. We included the following terms: (“Non-alcoholic Fatty Liver Disease” OR “Fatty Liver, Non-alcoholic” OR “Livers, Non-alcoholic Fatty” OR “Non-alcoholic Fatty Liver” OR “Liver, Non-alcoholic Fatty” OR NAFLD OR “Non-alcoholic Fatty Liver Disease” OR “Non-alcoholic Fatty Livers” OR “Steatohepatitis, Non-alcoholic”) AND (“Ramadan fasting” OR Ramadan OR “Recurrent circadian fasting” OR “Islamic fasting” OR “Ramadan diurnal fasting” OR “intermittent fasting” OR “Ramadan intermittent fasting” OR “Ramadan model of intermittent fasting” OR “Ramadan intermittent fasting” OR Ramadan OR RF OR intermittent OR “Intermittent prolonged fasting during Ramadan” OR “Ramadan fast”). We also manually searched the references of relevant reviews and articles. Two authors (Lin and Wu) conducted the search and in cases of uncertainty, they consulted with a third author (Huang).

### Inclusion criteria

Using the PICOS framework, we set the following inclusion criteria: (P) Populations: individuals with NALFD. (I) Interventions: Ramadan fasting. (C) Control: without Ramadan fasting. (O) Outcomes: Effects of Ramadan fasting on liver parameters, body weight, body composition, cardiovascular markers, glucose profiles, and inflammatory markers. (S) Study Types: Clinical studies, including cross-sectional studies, case-control studies, cohort studies, and randomized controlled trials (RCTs). We excluded editorials, duplicates, commentaries, conference abstracts, supplements, and case reports.

### Quality appraisal and data extraction

Various tools were utilized to evaluate study quality, depending on the specific study design. For randomized controlled trials (RCTs), we used the RoB2 tool. For non-RCTs, the ROBINS-I tool designed specifically for these types of studies was employed (36). The Newcastle-Ottawa Scale (NOS) was used to assess the quality of case-control and cohort studies (37). This scale comprises eight key questions addressing aspects such as participant selection, the comparability of study groups, and verification of exposure. Two authors (Lin and Wang) independently carried out the data extraction process. They collated the information into two pre-prepared tables, detailing the characteristics and main findings of the studies, including study year, design, mean age, mean BMI, study duration, main measured parameters, and body weight and composition, lipid profile, glucose and insulin metabolism, liver parameters, other outcomes.

## Results

### Literature search

**FIGURE 1:**
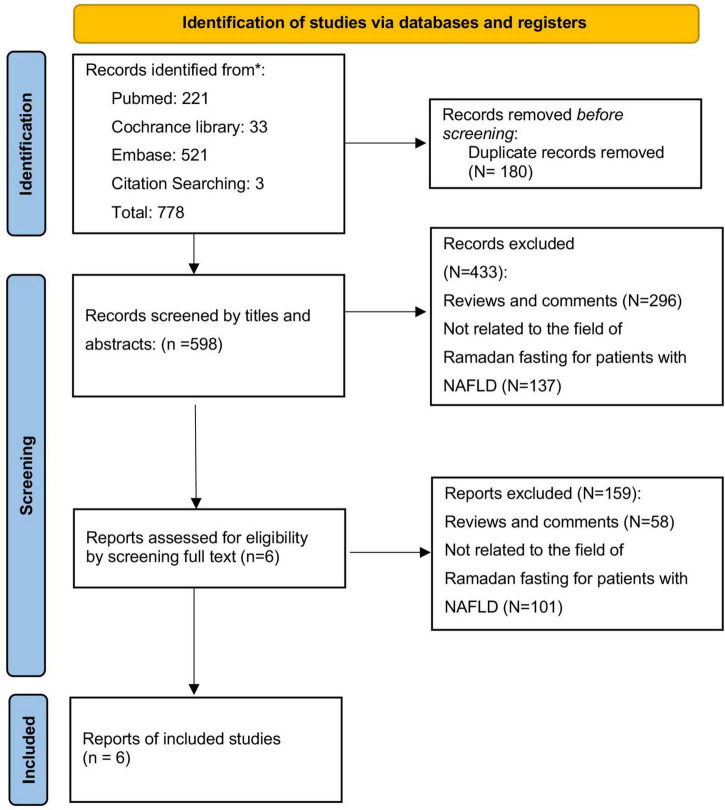
PRISMA 2020 flow diagram for new systematic reviews which included searches of databases and registers only.

Our initial database search yielded a total of 778 records. After removing duplicates, 598 records remained for screening by titles, abstracts, and full texts. Ultimately, we included six studies in the systematic review of five were prospective studies and one retrospective study (30–34, 38). The progression of the search and selection process is illustrated in Figure 1.

### Study and patient characteristics and the assessment of quality

A total of 397 participants were encompassed in the studies. Individual study sample sizes varied, with a range from 16 to 155 participants. The mean age spanned from 30.9 ± 2.42 to 51.8 ± 20.9 years, while the mean BMI ranged from 29.46 ± 4.52 to 37.03 ± 6.56. Each study had a duration of 4 weeks. Key measured parameters included body weight, body composition, cardiometabolic risk factors, glucose profiles, liver parameters, and inflammation markers. In the quality assessment, three studies (32, 33, 38) received a score of 9 and other three studies (30, 31, 34) were a score of 8 on the NOS scale and were thus classified as high quality. A detailed summary of the study characteristics and their quality assessments can be found in Tables 1, 2.

**TABLE 1:** The study characteristics.

References	Design	*N*	Mean age	Mean BMI	Study duration	Main measured parameters
Aliasghari (33)	The prospective study	102	37.59 ± 7.06	30.09 ± 4.49	4 weeks	Body weight, BMI, WC, Waist/Hip ratio, Fat mass, FBG, Insulin, HOMA-IR, IL-6, Hs-CRP
Arabi (34)	The prospective study	50	40.52 ± 10.90	31.38 ± 4.9	4 weeks	BMI, WC, Fat mass, Free fatty mass, SBP, DBP, ALT, AST, FBG, Insulin, TG, HDL, LDL
Badran (30)	The prospective study	16	46.03 ± 11.72	37.03 ± 6.56	4 weeks	BMI, FIB-4, cholesterol, triglycerides, HDL, LDL, cholesterol/HDL risk ratio
Mari (32)	The retrospective study	155	51.8 ± 20.9	36.7 ± 7.1	4 weeks	BMI, ALT, AST, Insulin, HOMA-IR, hs-CRP
Rahimi (38)	The prospective study	34	46.03 ± 11.72	29.46 ± 4.52	4 weeks	Body weight, BMI, ALT
Gad (31)	The prospective study	40	30.9 ± 2.42	30.9 ± 2.42	4 weeks	BMI, WC, Fat mass, FBG, Insulin, HOMA-IR, FIB-4, CAP, and LSM

**TABLE 2:** The quality assessments.

Items	Aliasghari (33)	Arabi (34)	Badran (30)	MARI (32)	Rahimi (38)	Gad (31)
**Selection (0–4 stars):**	4 stars	3 stars	3 stars	4 stars	4 stars	3 stars
Representativeness of the exposed cohort (1 star)	✩	✩	✩	✩	✩	✩
Selection of the non-exposed cohort (1 star)	✩	NA	NA	✩	✩	NA
Ascertainment of exposure (1 star)	✩	✩	✩	✩	✩	✩
Demonstration that the outcome of interest was not present at the start of the study (1 star)	✩	✩	✩	✩	✩	✩
**Comparability (0–2 stars):**	2 stars	2 stars	2 stars	2 stars	2 stars	2 stars
Comparability of cohorts on the basis of the design or analysis, controlled for the most important factor (1 star)	✩	✩	✩	✩	✩	✩
Control for any additional factor (a maximum of 1 star)	✩	✩	✩	✩	✩	✩
**Outcome (0–3 stars):**	3 stars	3 stars	3 stars	3 stars	3 stars	3 stars
Assessment of outcome (1 star)	✩	✩	✩	✩	✩	✩
Was the follow-up long enough for outcomes to occur (1 star)	✩	✩	✩	✩	✩	✩
Adequacy of follow-up of cohorts (1 star)	✩	✩	✩	✩	✩	✩
**Total scores**	**9**	**8**	**8**	**9**	**9**	**8**

Total scores (0–9 stars). Total scores are the sum total of the scores of selection, comparability, and outcome.

### The impact of Ramadan fasting for NAFLD

All six studies we reviewed investigated the effects of Ramadan fasting on body weight and composition (30–34, 38). While the majority showed that Ramadan fasting led to a decrease in body weight or an improvement in body composition, one study found no significant changes in these factors. Cardiometabolic risk factors were measured in four studies (30, 31, 33, 34), including lipid profiles, such as reductions in the levels of triglycerides, LDL, and total cholesterol, and an increase in HDL level. Three studies (30, 31, 33) found that Ramadan fasting had benefits for cardiometabolic risk while one not (32). Out of the five studies that assessed glucose parameters (30–34), four studies (30, 31, 33, 34) reported improvements in insulin resistance and sensitivity and a decrease in fasting blood sugar levels after Ramadan fasting. However, one study (32) noted an increase in fasting blood sugar levels post-fasting. Increased lipid and FBS levels may result from eating more high-carb and sugar foods and exercising less in the Ramadan period in this study. Liver parameters were a common focus across all studies. Of the five that investigated changes in ALT and AST post-fasting (30–32, 34, 38), four (30–32, 34) observed a decrease in these levels, but one study (38) found an increase in ALT levels, it may be due to individuals consume large amounts of food high in fat and sugar before dawn and sunset, which causes increased stress on liver parameters including ALT in this study. Mari et al. (32) reported significant decreases in NAFLD Fibrosis Score (NFS) (from 0.45 ± 0.25 to 0.23 ± 0.21, *P* < 0.05), fibrosis-4 (FIB4) scores (from 1.93 ± 0.76 to 1.34 ± 0.871, *P* < 0.05), and BARD scores (from 2.3 ± 0.98 to 1.6 ± 1.01, *P* < 0.05). Badran’s study (30) highlighted significant improvements in fibrosis markers, namely AST to platelet ratio index (APRI) and FIB-4 (*p* ≤ 0.05). Gad et al. (31) presented significant clinical improvements in FIB-4 (1.31 ± 0.26 to 1.24 ± 0.25, *p* < 0.05), controlled attenuation parameter (CAP) (from 318.52 ± 34.59 to 294.0 ± 20.34, *p* < 0.05), and liver stiffness measurement (LSM) (from 6.95 ± 1.62 to 6.59 ± 1.49, *p* < 0.05) after fasting. Inflammatory markers were evaluated in three studies, with each noting a decrease in C-reactive protein (CRP) levels post-Ramadan. Aliasghari’s study also identified a decrease in interleukin 6 (IL-6) levels after fasting. The primary findings of each study are summarized in Table 3.

**TABLE 3:** The primary findings of each study.

References	Body weight and composition	Lipid profile	Glucose and insulin metabolism	liver parameters	Other outcomes
Aliasghari (33)	Decreased BW	Decreased cholesterol level	decreased fasting blood glucose and plasma insulin	Decrease the level of steatosis	Decreased IL-6 and hs-CRP
Arabi (34)	No change in BMI, fat mass, fat-free	Increased HDL-C, TC, and triglycerides, Decreased Systolic blood pressure	Increased fasting blood glucose	Decrease ALT	NA
Badran (30)	Decreased BW and BMI	Decreased cholesterol level	decreased fasting blood glucose	Decreased Fib4 score and APRI score Decrease ALT	Decreased CRP
MARI (32)	Decrease BMI	NA	Decreased HOMA-IR	Decreased NFS score and BARD score, decrease ALT	Decreased CRP
Rahimi (38)	Decrease in weight gain	NA	NA	Increased ALT	NA
Gad (31)	Decrease BMI	Decreased cholesterol level, triglycerides, LDL cholesterol, total cholesterol, improve HDL cholesterol	Decreased fasting glucose level and HbA1c	Decreased liver steatosis and liver stiffness, Decrease ALT, FIB-4, CAP, and LSM	Decreased serum albumin and total protein

## Discussion

To the best of our knowledge, this study represents the first comprehensive systematic review that summarizes and analyzes all clinical studies examining the effects of Ramadan fasting on patients with NAFLD. In our systematic review, we included six studies, all of which were assessed as high quality. Our findings suggest that Ramadan fasting could serve as an effective dietary intervention for NAFLD patients, leading to improvements in body weight, liver parameters, lipid profiles, and inflammatory markers.

Ramadan fasting, an annual religious observance practiced by a significant portion of the global Muslim population, involves extended periods of abstention from food and fluid intake from dawn to sunset. Ramadan presents a significant departure from regular eating patterns and offers a distinctive, real-world context to investigate the effects of fasting and altered energy distribution on patients with NAFLD (39). This unique form of intermittent fasting has elicited considerable scientific interest in its potential therapeutic implications for metabolic disorders, notably NAFLD. Emerging research suggests that such protracted fasting intervals can induce beneficial metabolic shifts conducive to improved liver health. Specifically, Ramadan fasting has been associated with reductions in liver enzymes, signifying diminished hepatic stress and inflammation. Additionally, the fasting period can enhance insulin sensitivity, a paramount consideration given the integral role of insulin resistance in NAFLD pathogenesis. The potential for modest weight loss during Ramadan, coupled with shifts in lipid profiles toward a more cardioprotective phenotype, further underscores the prospective benefits for individuals with NAFLD. In total, the potential beneficial mechanisms of Ramadan fasting for patients with NAFLD include: Ramadan fasting is usually linked with weight loss, a decrease in total fat mass, and enhancements in cardiometabolic risk factors, including improvements in lipid profiles, blood pressure, and glycemic parameters; Ramadan fasting was shown to improve HOMA-IR and fasting glucose; Ramadan fasting may reduce the levels of inflammatory cytokines including CRP and IL-6, and oxidative stress; Ramadan fasting has significant improvements in alkaline phosphatase, AST, and bilirubin. In our systematic review, six studies with 397 patients with NAFLD were included. Collectively, these findings suggest that Ramadan fasting could serve as a promising non-pharmacological approach, complementing existing therapeutic strategies for NAFLD. However, the potential benefits may be offset by the customary, and sometimes excessive, unhealthy dietary intake during Iftar—the meal to break the fast. This could potentially worsen NAFLD pathophysiology. For example, in Rahimi’ study, they found that an increase in ALT after Ramadan, and in Arabi’s study, they found the increased lipid and FBS levels, since participants following a period of fasting is to eat large meals that are high in fats and sugars at the beginning and end of the day. This eating pattern can place significant stress on the liver as it works to metabolize the nutrients that have been ingested. The complex relationship between Ramadan fasting and NAFLD warrants further investigation to ensure that religious practices harmoniously align with optimal liver health. Our results are consistent with a recent study which evaluate the impact of IF on older patients with NAFLD based on Clinicaltrials.gov Registry. Ramadan fasting, as well as IF, may have potential benefits for patients with NAFLD (40).

Our systematic review has several limitations. Firstly, only six studies have investigated the effects of Ramadan fasting on patients with NAFLD. Secondly, there are no randomized controlled trials (RCTs) examining Ramadan fasting in the context of NAFLD. Thirdly, the sample sizes in the existing studies are relatively small, ranging from 16 to 155 subjects. In addition, geographic dietary habits during Ramadan may impact outcomes and weight reductions and may explain some of the contradictory results reported in this review. Investigating the effects of Ramadan fasting on the discussed parameters with a larger sample would provide more comprehensive insights. Future research would benefit from higher-quality studies featuring rigorous designs, longer-term interventions, and extended follow-up periods.

In conclusion, Ramadan fasting has potential as an effective dietary intervention for NAFLD. However, the existing body of research on the effects of Ramadan fasting for NAFLD patients is limited. To solidify our understanding of its benefits, we need further high-quality studies in this field.

## Data availability statement

The original contributions presented in the study are included in the article/supplementary material, further inquiries can be directed to the corresponding authors.

## Author contributions

XL: Data curation, Formal analysis, Methodology, Project administration, Supervision, Validation, Writing – original draft, Writing – review and editing. GW: Data curation, Formal analysis, Methodology, Project administration, Supervision, Validation, Writing – original draft. JH: Conceptualization, Funding acquisition, Investigation, Resources, Software, Visualization, Writing – original draft, Writing – review and editing.
